#  Comparative Efficacy of Zonisamide and Pregabalin as an Adjunctive Therapy in Children with Refractory Epilepsy

**Published:** 2015

**Authors:** Mohammad Mahdi TAGHDIRI, Mohammad Kazem BAKHSHANDEH BALI, Parvaneh KARIMZADEH, Mohammad Reza ASHRAFI, Seyed Hassan TONEKABONI, Mohammad GHOFRANI

**Affiliations:** 1 Pediatric Neurology Research Center, Shahid Beheshti University of Medical Sciences, Tehran, Iran; 2 Pediatric Neurology Center of Excellence, Department of Pediatric Neurology, Mofid Children Hospital, Faculty of Medicine, Shahid Beheshti University of Medical Sciences, Tehran, Iran; 3 Pediatrics Center of Excellence, Department of Pediatric Neurology, Children’s Medical Center, Tehran University of Medical Sciences, Tehran, Iran

**Keywords:** Epileptic children, Intractable Epilepsy, Antiepileptic drugs, Zonisamide, Pregabalin

## Abstract

**Objective:**

Approximately one third of epileptic children are resistant to anticonvulsant drugs. This study evaluates the effectiveness, safety, and tolerability of pregabalin as adjunctive therapy in epileptic children relative to Zonisamide.

**Materials & Methods:**

From April 2012 to November 2012,121 children were referred to Mofid Children’s Hospital with intractable epilepsy and enrolled in the study. The patients were divided into two groups (A and B) randomly. Group A was treated with Zonisamide and group B was treated with Pregabalin in addition to prior medication. We assessed seizure frequency and severity during a 4-week interval from the beginning of the drug treatment and compared the efficacy of each in these two groups.

**Results:**

Group A consists of 61 patients, 26 (42.6%) girls, and35 (57.4%) boys with an age range from 1.5 months–14 years (mean, 73.9± 44.04 months). Group B consists of 60 patients, 31(51.7%) girls, 29 (48.3%) boys with an age range from 6 months–16 years (mean, 71±42.9 months). Age, gender, seizure onset, seizure frequency, seizure type, and previous antiepileptic medications showed that there was no significant difference between the groups (P>0.05). Zonisamide and pregabalin reduced more than 50% of seizure intensity in 40.2%; 45.8% of patients also had a seizure frequency decline between35.8–44.4%, respectively and there was no significant superiority between these two novel anticonvulsants (P>0.05).

**Conclusion:**

In this survey both pregabalin and Zonisamide were impressive for seizure control in children with intractable epilepsy and well sustained with mild complications that were completely reversible.

## Introduction

Every year about 40 out of 100,000 children under 16 years of age are affected by epilepsy as one of the most widespread neurologic disorders worldwide (1). Approximately one third of epileptic children do not achieve complete seizure improvement in spite of equal or more two antiepileptic therapies, i.e. refractory epilepsy (2-3). Election of the most suitable anticonvulsant for an epileptic patient is problematic as the performance of the approved first-line treatments does not significantly differ. The primary evaluation proceeding for a new antiepileptic drug implicates its efficacy confirmation as an add-on therapy for a decline in seizure frequency in patients who have intractable seizures (4-5). Zonisamide (1,2 benzisoxazole- 3-methane sulfonamide) is a new production antiepileptic drug authorized as monotherapy and adjunctive therapy of intractable partial epilepsies. Zonisamide (ZNS) is effective in children with generalized epilepsies, notably for myoclonic seizures. Chemical organization (non-arylamine sulfonamide) and various manners of antiepileptic activity make ZNS incomparable to other antiepileptic drugs(AEDs). The main biochemical effect of ZNS is voltage linked sodium and T-type calcium channel blocking; however, glutamate related synaptic stimulation decline, gamma-aminobutyric acid (GABA) release from the hippocampus, neuronal hyper synchronization suppression, hydroxyl and oxide radicals elimination, and erythrocyte carbonic anhydrase deterrence are the other mechanisms. ZNS is absorbed rapidly and reaches peak plasma concentrations in 2–6 h after oral consumption. Additionally, it has high (95%) bioavailability (6-11). Pregabalin (PGB) has the structural equivalent of GABA but has no effects associated with GABA or GABA receptors(12).PGB is a selective high-propensity ligand for the α2-δ subunit of presynaptic voltage gated calcium channels. Intense binding to this channel diminishes calcium entrance at hyper excitable nerve terminations to lower the distribution of several neurotransmitters consisting of glutamate, norepinephrine, and substance P.A reduction of these neurotransmitters and neuronal excitability that this drug acts on leads to it as an anticonvulsant, analgesic, and anxiolytic efficacy(13-16).It has a linear dose–concentration curve that is among the most effectual dose span (150–600 mg/day) and extensively absorbed after oral dosing with 90% bio availability that after1 h reaches peak plasma concentrations. PGB is much more vigorous (2 to 18 fold) than gabapentin between all seizure types tested (17).Efficacy of PGB as an add-on therapy for patients with refractory partial epilepsies was evaluated through several controlled trials (18-19). 

As there is an insignificant amount of information regarding new AEDs utility in treatment of children with refractory epilepsy, the current assay was carried out to evaluate the effectiveness, safety, and tolerability of pregabalin as an adjunctive therapy relative to that of Zonisamide. 

## Materials & Methods

From April 2012 to November 2012,121 children were referred to Mofid Children’s Hospital with intractable epilepsy (failure of seizure recovery in spite of two or more antiepileptic drugs) and were enrolled in the study. Exclusion criterion was hypersensitivity to anticonvulsants and having a neurodegenerative disease. All parents endorsed a written informed consent. The initial assessment included history taking (seizure type, onset, etiology and frequency, period of the treatment, type of antiepileptic drug usage), general and neurologic physical examination, electroencephalography (EEG), and magnetic resonance imaging (MRI) performance. Then, the patients were divided into two groups randomly (A and B).Group A was treated with Zonisamide while patients in group B received Pregabalin. We treated group A with capsules of ZNS (2–12 milligram per kilogram daily) as group B were treated with capsules of PGB (5–15 milligram per kilogram daily)divided in two or three doses in addition to prior medication. We assessed seizure frequency, severity, and duration during the 4 week interval from the beginning of treatment and compared the efficacy of each drug in the two groups. During follow-ups, seizure frequency or severity reduction equal and greater than 50%istermedas a response to the drug. A paired sample T-test, Z-test, and chi-square have been used in the statistical analysis. All of the ethical perspectives of this study have been confirmed by the Ethics Committee of Shahid Beheshti University of Medical Sciences. This study was registered in the Iranian Registry of Clinical Trial (IRCT) as IRCT2012091210508N4. 

## Results

In this study, 137 patients were enrolled and121 patients reached the final stage. Table 1 lists the details of patient specifications. Group A consists of 61 patients, 26 (42.6%) girls, 35 (57.4%) boys with an age range of1.5 months–14 years (mean, 73.9± 44.04 months) (Table.1). Group B consists of 60 patients, 31(51.7%) girls, 29 (48.3%)boys with an age range from 6 months to 16 years (mean, 71±42.9 months) (Table.1).The mean age of seizure onset in group A and group B was 27.1±28.1 and 23.15±24.3 months, respectively. The mean seizure frequency per day in group A was 5.43±7.21 with attacks ranging from 3 every month to 50 daily. The mean seizure frequency per day in group B was 4.65±3.77 with attacks ranging from 3 every month to 20 daily. The most common seizure form in group A was generalized tonic clonic, partial, and myoclonic seizures in 17 (27.9%),16(26.3%),and 11(18%)children, respectively (Table1).The most common seizure form in 22 (36.7%),16(26.3%),and 8(13.3%)children of group B was generalized tonic clonic, partial, and mixed type seizures, respectively (Table1).According to seizure etiology 26(42.62%),24 (39.34%), and 11 (18%) patients of group A were classified as idiopathic, cryptogenic, and symptomatic epilepsy, respectively (Table1).Idiopathic, cryptogenic, and symptomatic epilepsy were seizure etiology of 23(38.3%),30 (49.18%), and 7 (11.7%) patients of group B, respectively (Table1).The EEG findings were normal, mild, moderate, and severely abnormal in 4 (6.57%), 18 (29.5%), 24 (39.34%), and 15 (24.6%)children of group A, respectively(Table 1).The EEG findings in 4 (6.57%), 17 (28.3%), 25 (41.66%), and 14 (23.33%)children of group B were normal, mild, moderate, and severely abnormal, respectively(Table 1). Patients of group A had used a minimum 3 and maximum of 15 anti-seizure medications, the average number of 7.11±2.97 prior to this study. Patients of group B had used minimum 3 and maximum of 13 anti-seizure medications, the average number of 7.28±2.92 prior to this study. Age, gender, seizure onset, seizure frequency, seizure type, and previous antiepileptic medications indicated there were no significant differences between these two groups (P>0.05). 

**Fig 1:**
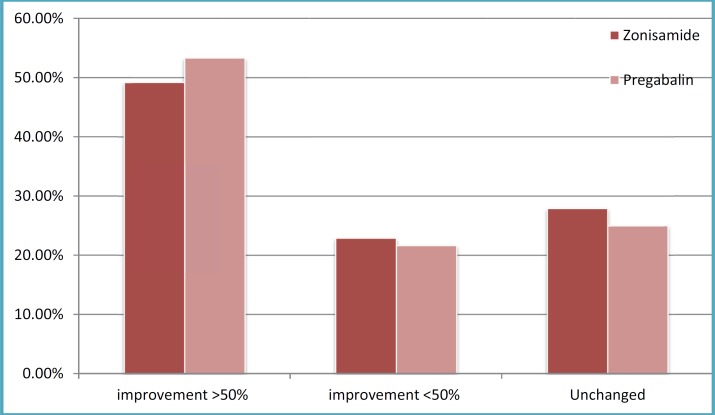
Seizure reduction in the first month follow-up

After one and six months of ZNS usage, daily seizure frequency decreased to 2.95±3.54as 45.7% reduction and 3.73±3.5 as 31.3% reduction, respectively. PGB after one and six months reduced seizure frequency up to 2.41±2.38as a 48% reduction and 2.75±2.38 as a 40.86% reduction respectively. According to improvements in seizure severity and duration after one month, 17(27.9%),30(49.2%), and 14 (22.9%) patients of group A toward 15(25%),32(53.33%), and 13 (21.66%) patients of group B had no change, greater than 50% recovery, and reduction less than 50%, respectively (Figure1). Also,25(41%),19 (31.1%),14(23.9%), and 3(4.9%) patients of group A compared to 18(30%),23 (38.33%),17(28.3%), and 2(3.3%) patients of group B were not changed, reduced greater than 50%, had reduction lower than 50%, and had worsened in the 6 months follow-up, respectively(Figure1).There was no significant difference between these two groups in view of seizure frequency, duration, and severity reduction after one(P=0.591) and six (P=0.607) months reviews. First month reviews showed that infantile spasms (85.7%), generalized tonic clonic (58.8%), and myoclonic (54.5%) seizures compared to partial (70.2%), tonic (60%), and generalized tonic clonic (40.9%) were the types of seizures with the highest response to ZNS and PGB respectively. The highest response to ZNS and PGB were in seizure types as follows: Myoclonic (54.5%), infantile spasms (47.1%), and generalized tonic clonic (42.9%) versus partial (53.2%), tonic (50%), and atonic (40%) during six-month review, respectively. There was no seizure response to mixed type and myoclonic seizures by ZNS and PGB, respectively, within the first or six months assay. 

## Discussion

This study showed that ZNS and PGB reduced greater than 50% of the seizure intensity in 40.2% and 45.8% of patients also had seizure frequency declinesof35.8% and 44.4%, respectively in which there was no significant superiority between these two novel anticonvulsants. Brodie et al, Sackellares et al, Faught et al, and Schmidt et al reported a response rate (greater than 50% seizure reduction) of 28–47% with ZNS as an add-on therapy for intractable epilepsy through four clinical studies (20). ZNS effectiveness in the current assay is in agreement with Coppola et al and Stephen who declared a frequency decline of greater than 50% between 48.7–39% of patients, respectively(21-22).No preference between these two drugs that we found is different from Claudia B et al and Yuen et al who estimated that with a ZNS retention rate of 31% compared to 24% for PGB after six months period (23-24).In our review, the efficacy of both drugs has been diminished after six months of treatment towards one month of consumption. Loscher et al has observed that the efficacy of antiepileptic drugs decreased over time due to drug receptor sensitivity decreases that led to pharmaco dynamic tolerance that can further lead to loss of drug function completely or could interfere with other anticonvulsant activity via cross tolerance(25).Similar to our results, Arroyo et al stated that 43.5% of patients with refractory epilepsy responded to PGB therapy. Furthermore, this study found a dose-dependent response to this drug at the higher dose equal to 600 mg per day was quite effective(15).Terence et al examined the effects of lamotrigine compared with pregabalin on refractory partial seizures in a 17-week treatment period and concluded that PGB reduced greater than 50% of seizure intensity in 35.5% of cases toward the lamotrigine efficacy in 21.4% of patients(26).Lee et al observed seizure declines in 46.2% of patients after 13 weeks of PGB treatment, which confirmed our result (27). Elger et al detected PGB antiepileptic efficacy more than our results who treated patients with a constant dose of PGB (600mg/day) and later 12 weeks found 49.3% seizure improvement (19).This discrepancy may be due to a wide spectrum of PGB dosage (5-15mg/kg/day), which takes a few weeks to achieve a fixed amount of the drug’s availability in patients. Pradeep found a significant PGB induced reduction in the frequency and severity of seizures of up to 56% among patients with various seizure types and those who had previously not responded to gabapentin had no response to PGB(28). Our study showed that three patients who had used gabapentin previously did not respond to PGB against Pradeep’s results. The highest response to ZNS in our case was obtained in seizure types of infantile spasms (66.4%), myoclonic (54.5%), and generalized tonic clonic (50.9%) with no effect on mixed type seizures. Partial seizures (61.7%), tonic (55%), and generalized tonic clonic (40.9%) were the seizure kinds with the highest response to PGB in our patients with no effect on myoclonic seizure. PGB and ZNS had complication rate of 18.33–16.4%, respectively. Decreased appetite, impaired speech, ataxia, sigh, visual disturbances, hallucinations, and dizziness versus increased appetite, increased urination, hallucinations, and headache have been the most prevalent side effects of ZNS and PGB, respectively. All of our patients’ complications occurred with ZNS and PGB dose greater than or equal to 200vs 150 mg/day in patients over 6vs 7 years of age, respectively. The incidence of ZNS complications in the current assay is insignificant compared to Tan et al (43.9%), Stephen (28.6%) and Claudia et al(58%) detections. Since, usually PGB has been used in the treatment of adults with epilepsy that requires at least 300 mg/day. Its complications incidence in n Lee et al (64.7%), Terence et al (50%), and Elger et al (32.8%) surveys were greater than our results. Weight gain was the most common side effect of PGB in our patients and was also Pradeep, Arroyo et al, and Terence et al reported 5–7% weight enhancement among 24–50% of those who took it. 

**Table 1:** Summary of Patient Characteristics

Characteristic	Zonisamide. Group 2-12mg/kg/day (*N *= 61)	Pregabalin. Group 5-15mg/kg/day (*N *= 60)	Characteristic	Zonisamide. Group 2-12mg/kg/day (*N *= 61)	Pregabalin. Group 5-15mg/kg/day (*N *= 60)
**Gender**			Seizure frequency		
**Male** **Female**	35 (57.4%) 26 (42.6%)	29 (48.3%) 31 (51.7%)	Mean (SD) Range	5.43 ± 7.2 0.3 - 50	4.65 ± 3.77 0.1 - 20
**Age**			Seizure.onset		
**Mean (SD)** **Range**	73.9±44m1.5m to 14 y	71±42.89m6 m to 16 y	Mean (SD) Range	27.1±28.1 m 3 D - 11 y	23.2±24.3 m 15 D - 10 y
**Seizure.type**			MRI		
**Tonic clonic** **Simplepartial** **Complexpartial** **Total partial** **Infantile spasm** **Myoclonic** **Tonic** **Atonic** **Absence** **mixed**	17 (27.9%) 4 (6.6%) 12 (19.7%) 16 (26.3%) 7 (11.5%) 11 (18%) 2 (3.3%) 0 (0%) 0 (0%) 8 (13.1%)	22 (36.7%) 3 (5%) 13 (21.7%) 16 (26.3%) 3 (5%) 3 (5%) 5 (8.3%) 2 (3.3%) 1 (1.7%) 8 (13.3%)	Normal Atrophy PVL Tuberous sclerosis Migrationaldisorder M. temporal sclerosis Cortical dysplasia Callosal dysgenesis Encephalomalacia Basal ganglia Calcification Focal lesion	19 (31.1%) 17 (27.9%) 10 (19.4%) 3 (4.9%) 4 (6.6%) 2 (3.3%) 2 (3.3%) 2(3.3%) 0 (0%) 1 (1.6%) 0 (0%) 1 (1.6%)	20 (33.3%) 16 (26.7%) 5 (8.3%) 2 (3.3%) 3 (5%) 6 (10%) 2(3.3%) 0 (0%) 1 (1.6%) 1 (1.6%) 2 (3.3%) 3 (5%)
**EEG.quality**			EEG.waves		
**Normal** **Mild abnormal** **Moderate abnormal** **Severe abnormal**	4 (6.6%) 18 (29.5%) 24 (39.34%) 15 (24.6% )	4 (6.57%) 17 (28.3%) 25 (41.66%) 14 (23.33% )	Spike High voltage slow wave Hypsarrhythmia Sharp wave Burst suppression	23(37.7%) 16(26.22%) 9(14.57%) 6(9.83%) 3(4.9%)	29(48.33%) 12(20%) 5(8.33%) 8(13.33%) 2(3.33%)
**First month follow-up**			Six-month follow-up		
**Unchanged** **Improvement** **75-99% reduction** **50-75% reduction** **25-50% reduction** **<25% reduction**	17(27.9%) 9(14.8%) 8(13.1%) 13(21.3%) 8(13.1%) 6(9.1%)	15(25%) 11(18.3%) 8(13.33%) 13(21.3%)10(16.66%) 3(5%)	Unchanged Improvement 75-99% reduction 50-75% reduction 25-50% reduction <25% reduction worsening	25(41%) 9(14.8%) 1(1.6%) 9(14.8%) 7(11.5%) 7(11.5%) 3(4.9%)	18(30%) 11(18.3%) 6(10%) 6(10%) 9(15%) 8(13.3%) 2(3.3%)

M= month, y= years, MRI=Magnetic resonance imaging, PVL=Periventricular leukomalacia, HVSW= High voltage slow waves

**Fig 2:**
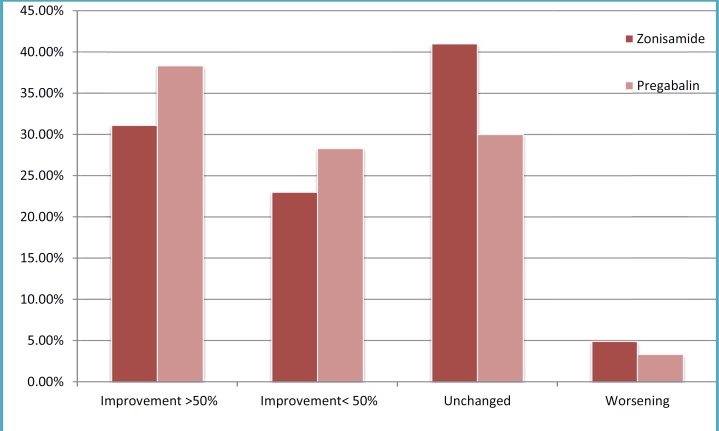
Seizure reduction in the six-month follow-up

**In conclusion, **in this survey both pregabalin and Zonisamide were impressive for seizure control in children with intractable epilepsy and well sustained with mild complications that were completely reversible. 

## References

[1] Kwan P, Brodie MJ (2000). Early identification of refractory epilepsy. N Engl J Med.

[2] Berg AT, Shinnar S, Levy SR, Testa FM, Smith-Rapaport S, B (2001). Early development of intractable epilepsy in children: a prospective study. Neurology.

[3] Comﬁeld PR, Camﬁeld CS, Swaiman KF, Ashwals, Ferriero DM (2006). pediatric epilepsy: An overview. Pediatric Neurology: principles and practice.

[4] Porter RJ, Baulac M, Nohria V (2010). Clinical development of drugs for epilepsy: a review of approaches in the United States and Europe. Epilepsy Res.

[5] Kwan P, Brodie MJ (2003). Clinical trials of antiepileptic medications in newly diagnosed patients with epilepsy. Neurology.

[6] LepiSobieszek G, Borowicz KK, Kimber-Trojnar Z, Małek R, Piskorska B, Czuczwar SJ (2003). Zonisamide: a new antiepileptic drug. Pol J Pharmacol.

[7] kk IE (2004). Zonisamide: chemistry, mechanism of action, and pharmacokinetics. Seizure.

[8] Ohtahara S (2006). Zonisamide in the management of epilepsy Japanese experience. Epilepsy Res.

[9] Baulac M (2006). Introduction to zonisamide. Epilepsy Res.

[10] Heo K, Lee BI, Yi SD, Cho YW, Shin DJ, Song HK, Kim OJ, Park SP, Kim SE, Kim SH, Lee JH, Kim KS, Lee SJ (2012). Short-term efﬁ and safety of zonisamide as adjunctive treatment for refractory partial seizures: A multicenter open-label single-arm trial in Korean patients. Seizure.

[11] Hwang H, Kim KJ (2008). New antiepileptic drugs in pediatric epilepsy. Brain Dev.

[12] Fehrenbacher JC, Taylor CP, Vasko MR (2003). Pregabalin and gabapentin reduce release of substance P and CGRP from rat spinal tissues only after inflammation or activation of protein kinase C. Pain.

[13] Field MJ, Cox PJ, Stott E, Melrose H, Offord J, Su T-Z, Bramwell S, Corradini L, England S, Winks J, Kinloch RA, Hendrich J, Dolphin AC, Webb T, Williams D (2006). Identification of the α2-δ-1 subunit of voltage-dependent calcium channels as a molecular target for pain mediating the analgesic actions of pregabalin. PNAS.

[14] Taylor CP, Angelotti T E (2007). Pharmacology and mechanism of action of pregabalin: The calcium channel α2-δ (alpha2- delta) subunit as a target for antiepileptic drug discovery. Epilepsy Res.

[15] Ben-Menachem E (2004). Pregabalin pharmacology and its relevance to clinical practice. Epilepsia.

[16] Taylor CP, Angelotti T E (2007). Pharmacology and mechanism of action of pregabalin: the calcium channel alpha2delta (alpha2-delta) subunit as a target for antiepileptic drug discovery. Epilepsy Res.

[17] French JA, Kugler AR, Robbins JL, Knapp LE, Garofalo EA (2003). Dose-response trial of pregabalin adjunctive therapy in patients with partial seizures. Neurology.

[18] Elger CE, Brodie MJ, Anhut H, Lee CM, Barrett JA (2005). Pregabalin add-on treatment in patients with partial seizures: a novel evaluation of flexible-dose and fixeddose treatment in a double-blind, placebo controlled study. Epilepsia.

[19] Arroyo S, Anhut H, Kugler AR, Lee CM, Knapp LE, Garofalo EA S (2004). Pregabalin add-on treatment: a randomized, double-blind, placebo-controlled, doseresponse study in adults with partial seizures. Epilepsia.

[20] Baulac M IE (2007). Efﬁcacy and safety of adjunctive Zonisamide therapy for refractory partial seizures. Epilepsy Research.

[21] Stephen LJ, Kelly K, Wilson EA, Parker P, Brodie MJ (2010). A prospective audit of adjunctive zonisamide in an everyday clinical setting. Epilepsy Behav.

[22] Baulac M, Leon T, O’Brien TJ, Whalen E, Barrett J (2010). A comparison of pregabalin, lamotrigine, and placebo as adjunctive therapy in patients with refractory partial-onset seizures. Epilepsy Research.

[23] Lee BI, Yi S, Hong SB, Kim MK, Lee SA, Lee SK, Shin DJ, Kim JM, Song HK, Heo K, Lowe W, Leon T (2009). Pregabalin add-on therapy using a ﬂexible, optimized dose schedule in refractory partial epilepsies: A double blind, randomized, placebo-controlled, multicenter trial. Epilepsia.

[24] Modur PN, Milteer WE (2008). Adjunctive pregabalin therapy in mentally retarded, developmentally delayed patients with epilepsy. Epilepsy & Behavior.

[25] Coppola G, Grosso S, Verrotti A, Parisi P, Luchetti A, Franzoni E, Mangano S, Pelliccia A, Operto FF, Iannetti P, Curatolo P, Balestri P A (2009). Zonisamide in children and young adults with refractory epilepsy: An open label, multicenter Italian study. Epilepsy Research.

[26] Tan HJ, Martland TR, Appleton RE R (2010). Effectiveness and tolerability of zonisamide in children with epilepsy: A retrospective review. Seizure.

[27] Catarino CB, Bartolini E, Bell GS, Yuen AW, Duncan JS, Sander JW (2011). The long-term retention of zonisamide in a large cohort of people with epilepsy at a tertiary referral centre. Epilepsy Research.

[28] Loscher W, Schmidt D (2006). Experimental and clinical evidence for loss of effect (tolerance) during prolonged treatment with antiepileptic drugs. Epilepsia.

